# Gene expression in BMPR2 mutation carriers with and without evidence of Pulmonary Arterial Hypertension suggests pathways relevant to disease penetrance

**DOI:** 10.1186/1755-8794-1-45

**Published:** 2008-09-29

**Authors:** James West, Joy Cogan, Mark Geraci, Linda Robinson, John Newman, John A Phillips, Kirk Lane, Barbara Meyrick, Jim Loyd

**Affiliations:** 1 Department of Medicine, Vanderbilt University Medical Center, Nashville, Tennessee, USA; 2 Department of Genetics, Vanderbilt University Medical Center, Nashville, Tennessee, USA; 3 Department of Pathology, Vanderbilt University Medical Center, Nashville, Tennessee, USA; 4 Division of Pulmonary Sciences and Critical Care Medicine, University of Colorado Health Sciences Center, Denver, Colorado, USA

## Abstract

**Background:**

While BMPR2 mutation strongly predisposes to pulmonary arterial hypertension (PAH), only 20% of mutation carriers develop clinical disease. This finding suggests that modifier genes contribute to FPAH clinical expression. Since modifiers are likely to be common alleles, this problem is not tractable by traditional genetic approaches. Furthermore, examination of gene expression is complicated by confounding effects attributable to drugs and the disease process itself.

**Methods:**

To resolve these problems, B-cells were isolated, EBV-immortalized, and cultured from familial PAH patients with BMPR2 mutations, mutation positive but disease-free family members, and family members without mutation. This allows examination of differences in gene expression without drug or disease-related effects. These differences were assayed by Affymetrix array, with follow-up by quantitative RT-PCR and additional statistical analyses.

**Results:**

By gene array, we found consistent alterations in multiple pathways with known relationship to PAH, including actin organization, immune function, calcium balance, growth, and apoptosis. Selected genes were verified by quantitative RT-PCR using a larger sample set. One of these, CYP1B1, had tenfold lower expression than control groups in female but not male PAH patients. Analysis of overrepresented gene ontology groups suggests that risk of disease correlates with alterations in pathways more strongly than with any specific gene within those pathways.

**Conclusion:**

Disease status in BMPR2 mutation carriers was correlated with alterations in proliferation, GTP signaling, and stress response pathway expression. The estrogen metabolizing gene CYP1B1 is a strong candidate as a modifier gene in female PAH patients.

## Background

Pulmonary arterial hypertension (PAH) is a lethal disorder characterized by pulmonary vascular constriction and remodeling leading to progressively worsening right ventricular hypertrophy, and eventually right heart failure. The familial form (FPAH) is usually caused by mutations in the type 2 receptor for the BMP pathway, BMPR2 [1-3].

The lifetime risk of developing PAH in a BMPR2 mutation carrier is less than 20%[4]. This suggests the need for environmental or genetic modifiers for full expression of the disease. Understanding modifier genes would both clarify the molecular etiology of the disease, which is obscured by the myriad of BMPR2 functions, and allow the FPAH risk to be refined for asymptomatic individuals that carry BMPR2 mutations. The search for these modifiers, which has been ongoing for several years, is complicated by many factors[5,6]. For example, since the modifier alleles are likely to be common variants, traditional genetic approaches are problematic, since the same allele could be associated with more than one line of inheritance within a family tree[7]. This is further complicated by the possibility that predisposition to disease within BMPR2 mutation carriers is caused by a confluence of factors rather than a single modifier gene, even within a single family.

Attempts to find contributing genes through gene array approaches have been tried, using lung tissue from transplant patients and circulating cells[8,9]. Interpretation of those data is made difficult by several factors. First, the majority of genes dysregulated are likely to be caused by the presence of end-stage disease. Second, drug effects cannot properly be controlled, and can be quite large. Finally, there is a large background in gene expression differences caused by the diverse background genetics of the patients. Finding direct evidence of modifier gene or even mutation effects within all of these sources of experimental noise is essentially impossible.

In order to overcome these issues, in this study we have used patient-derived lymphoblastoid cell lines to hunt for modifier genes. Lymphoblastoid cell lines, made by EBV-immortalizing B cells, resolve many of these issues. While culturing the cells introduces its own alterations in gene expression, these alterations are uniform across samples. Lymphoblastoid lines have been used successfully in gene expression studies for disease processes as diverse as schizophrenia, drug resistance, autism, and asthma[10-13]. Moreover, culturing the cells removes them from both the disease milieu and drug effect. While other types of cells may have changes in differentiation caused by the disease state, this is not true of B cells, whose lineage commitment is considered to be unidirectional and irreversible under physiologic conditions[14,15]. Finally, in order to minimize variation caused by genetic background we derived all of our lines from within the same extended family. An additional advantage is that we are directly measuring differences in baseline gene expression, without regard for either the polymorphism that caused them or their hereditary origin.

In this study, we use Affymetrix arrays to compare gene expression in BMPR2 mutation carriers that are either asymptomatic carriers (unaffected) or who have PAH (affected). We confirmed selected differentially expressed genes using quantitative RT-PCR on lines derived from a larger number of patients as well as from family members without mutation (non-carriers).

We found several broad pathways with differential expression between affected and unaffected BMPR2 mutation carriers, including stress response, actin organization/g-protein, calcium balance, and cell-cycle related genes. Analysis of overrepresented gene ontology groups suggests that it is pathway-specific, not gene-specific changes that are associated with increased risk.

## Methods

### Subjects

Ethylenediaminetetraacetic acid (EDTA) anticoagulated blood was collected from twenty individuals within one heavily affected Tennessee family with a reported mutation in BMPR2 (exon 3 T354G) (Table 1) [16]. Five of these individuals had hemodynamic evidence of FPAH (age, 15–39 yr; 3 females, 2 males); seven unaffected individuals carrying BMPR2 mutations had no evidence of PAH (age, 48–88 yr; 5 females, 2 males). Blood was also obtained from eight spouses within the family, but not in the bloodline, as control subjects (age, 36–67 yr; 5 females, 3 males).

**Table 1:** Subject Data

**Registry #**	**Gender**	**Phenotype**	**Age at Draw**	**Age at Diagnosis**	
1218	F	Non-carrier	41		
1217	F	Non-carrier	36		
1741	F	Non-carrier	61		
1688	F	Non-carrier	56		
1729	F	Non-carrier	67		
1744	M	Non-carrier	53		
1569	M	Non-carrier	65		
1227	M	Non-carrier	39		

1745	F	Unaffected Carrier	51		
1731	F	Unaffected Carrier	57		
1746	F	Unaffected Carrier	48		
176	F	Unaffected Carrier	88		*
172	F	Unaffected Carrier	65		*
1742	M	Unaffected Carrier	59		
180	M	Unaffected Carrier	66		*

1727	F	Affected	15	13	
723	F	Affected	35	29	*
264	F	Affected	17	9	*
266	M	Affected	39	26	*
186	M	Affected	29	24	*

Asymptomatic status was confirmed in each individual by echocardiography at the time of blood draw. Only unaffected carriers over the age of 60 were used for gene array experiments, since they had the least probability of later developing later disease, and would still have substantial difference in age of onset if they did harbor occult disease. Unaffected carriers are on average second degree relatives to each-other; they were on average sixth degree relation to FPAH patients. FPAH patients were on average eighth degree relations to each-other (they are 3^rd ^cousins, 3^rd ^cousins once removed, or 2^nd ^cousins once removed). The close relation in the unaffected carriers is coincidence; there are unaffected carriers in many branches of the family tree, but not that were currently available for blood draw and in the correct age range.

An additional 24 blood samples were collected from 3 additional families for confirmatory studies related to CYP1B1 expression. These include a family with an exon 4–5 deletion (3 non-carriers, 3 affected and 2 unaffected mutation carriers), a family with an exon 9 deletion (1 non-carrier, 3 affected and 3 unaffected mutation carriers), and a family with an exon 9 frameshift mutation (2 non-carriers, 2 affected and 5 unaffected mutation carriers).

The study was approved by the institutional review board at Vanderbilt University Medical Center, and written, informed consent was obtained from all subjects included in the study. Unique identifiers to conceal identity were assigned to the samples before their receipt in the laboratory.

### Lymphoblastoid Lines

Lymphocytes were isolated from anticoagulated whole blood within 48 hrs of collection and exposed to Epstein-Barr Virus (EBV) to induce cell immortalization. Two ml blood was diluted with 2 ml PBS, layered on top of 3 ml of Lympho Separation Medium (MP Biomedicals) and centrifuged for 10 minutes at 1,000 × g at room temperature. Using a Pasteur pipet, the lymphocytes were removed from the serum/Lympho Sep Media interface, washed in 10 ml PBS and then resuspended in 3 ml lymphoblast media (RPMI 1640 media containing L-glutamine, and 20% fetal bovine serum) containing 2 μg/ml cyclosporine. The lymphocytes were then infected with 3 ml Epstein-Barr virus (EBV) and transferred to a T-25 vent capped flask. The cells were incubated at 37°C/5% CO2 and fed weekly with lymphoblast media + cyclosporine until signs of growth occurred.

### Affymetrix Arrays

RNA was isolated from lymphocytes using a Qiagen RNeasy mini kit (Valencia, CA). First and second strand complimentary DNA was synthesized using standard techniques. Biotin-labeled antisense complimentary RNA was produced by an in vitro transcription reaction. Human Genome U133 Plus 2.0 microarrays (Affymetrix, Foster City, CA) were hybridized with 20 μg cRNA. Target hybridization, washing, staining, and scanning probe arrays were done following an Affymetrix GeneChip Expression Analysis Manual. All array results have been submitted to the NCBI gene expression and hybridization array data repository (GEO, ), as series GSE10767.

### Array Analysis

Affymetrix Cel files were loaded into dChip array analysis software[17]. Overall signal strength from arrays was normalized to the median array, and expression levels determined using the perfect match/mismatch (PM/MM) algorithm. Differentially expressed genes were determined using a 95% probability of a minimum 1.4× change and a minimum absolute difference of 150 (arbitrary units). Using random reassignment of group identity, this produced a median 17.2% false discovery rate, a reasonable compromise between sensitivity and specificity[18]. These requirements in themselves result in a list of genes in which there is no overlap in expression between groups. For every gene in the list, every affected mutation carrier has higher expression than every unaffected mutation carrier, or every affected mutation carrier has lower expression than every unaffected mutation carrier. Every gene thus has a p < .05 for significance by Wilcoxon rank-sum test.

Gene ontology was determined using the Classify Genes tool within dChip, with gene ontology files downloaded from the Gene Ontology Consortium , and were grouped for the purposes of this study by biological process (rather than molecular function or cellulcar component) classification[19,20]. Genes which lacked biological process annotation in the database were assigned to a group through a brief literature review. Most genes fall into several gene ontology groups. Thus, the selection of group for each gene was somewhat arbitrary. However, in the case of the genes dysregulated in this study, different choice of group would primarily shift genes between groups rather than create new groups. For instance, matrix genes can be involved in vascular contractility, stress response, and growth. Actin organization and G-proteins can be involved in both contractility and lymphocyte recruitment. Thus, the groups of genes identified are for the most part tightly interrelated in function, with functions directly related to the etiology of PAH.

### Quantitative RT-PCR

Primers were designed using Primer3 from sequences downloaded from Genbank, with primers tested for specificity by BLAST  ([21]. Primer sequences are listed in Table 2. Total RNA was made from lymphoblastoid lines independently of that used for arrays, also using a Qiagen RNeasy mini kit (Valencia, CA). First strand cDNA was made from 1 μg total RNA using a QuantiTect^® ^Reverse Transcription Kit (Qiagen, Valencia, CA). Quantitative real-time PCR was performed using a total reaction volume of 25 μl, containing 5 μl of diluted cDNA, 12.5 μl iTaq SYBR Green Supermix with ROX (BioRad Laboratories, Hercules, CA) and 0.03 μl of each oligonucleotide primer (250 μM). PCR was carried out in a 7300 Real Time PCR System (Applied Biosystems, Foster City, CA), using 40 cycles of 95°C for 15 seconds followed by 60°C for 1 minute with a ten minute 95°C initial soak. Each measurement was made in triplicate and expressed relative to the detection of the standard β-actin.

**Table 2:** Primer Sequences

Gene	Forward	Reverse	Product Size
B-Actin	GGA TGC CTC TCT TGC TCT G	GTC TTC CCC TCC ATC GTG	106 bp
CYP1B1	AAC GTA CCG GCC ACT ATC AC	ACG ACC TGA TCC AAT TCT GC	137 bp
NR2F2	CCA AGA GCA AGT GGA GAA GC	AGG CAT CTG AGG TGA ACA GG	92 bp
PRKCH	TAT TCG ATG TCA AGC GAA CG	ATA TTT CCG GGT TGG AGA CC	96 bp
RHOC	GAG AGC TGG CCA AGA TGA AG	GCA CTC AAG GTA GCC AAA GG	92 bp
SEPT10	CAT GAG TTC CAT GGT GAA CG	GCT CAA ATT TGG CCT GTA GC	124 bp
TWSG1	AAT GTT CAC GCG CCT TAT TC	AAC CAG CGA TAT TTG GAT GC	128 bp

### Statistics

Confidence intervals for fold changes in array analysis are determined by algorithms internal to dChip, as previously described[17]. These functionally result in this case in genes for which difference between affected and unaffected groups is also p < .05 by Wilcoxon signed-rank test.

Other statistical analyses were performed using the JMP program (SAS, Cary, NC). Comparisons for quantitative PCRs comparing three groups (non-carrier, unaffected, affected) were performed using Kruskal-Wallis, although results are similar using ANOVA. Analysis for sex-specific differences in carriers was performed using two-way ANOVA on log transformed values, with comparisons between individual values by Tukey's HSD post-hoc. Analyses of overrepresentation of gene ontology groups was performed by fisher's exact test, and overrepresentation of genes within those groups were performed using the one sample z test. While a substantial body of literature is arising suggesting alternate and potentially more powerful methods of determining overrepresentation of gene ontology groups, the field still seems to be in flux and so we have continued to rely on Fisher's exact test for this purpose[22].

## Results

### BMPR2 mutation carriers with disease show alteration in multiple PAH-related pathways compared to unaffected carriers

RNA derived from lymphoblastoid lines from four affected and three unaffected BMPR2 mutation carriers (marked with * on Table 1) were used to probe Affymetrix U133 Plus 2.0 gene expression arrays. These included every lymphoblastoid line from affected carriers within the family available at the time (one additional line was derived later), and every line from unaffected carriers over the age of sixty (who were thus most likely to remain unaffected).

**Figure 1:**
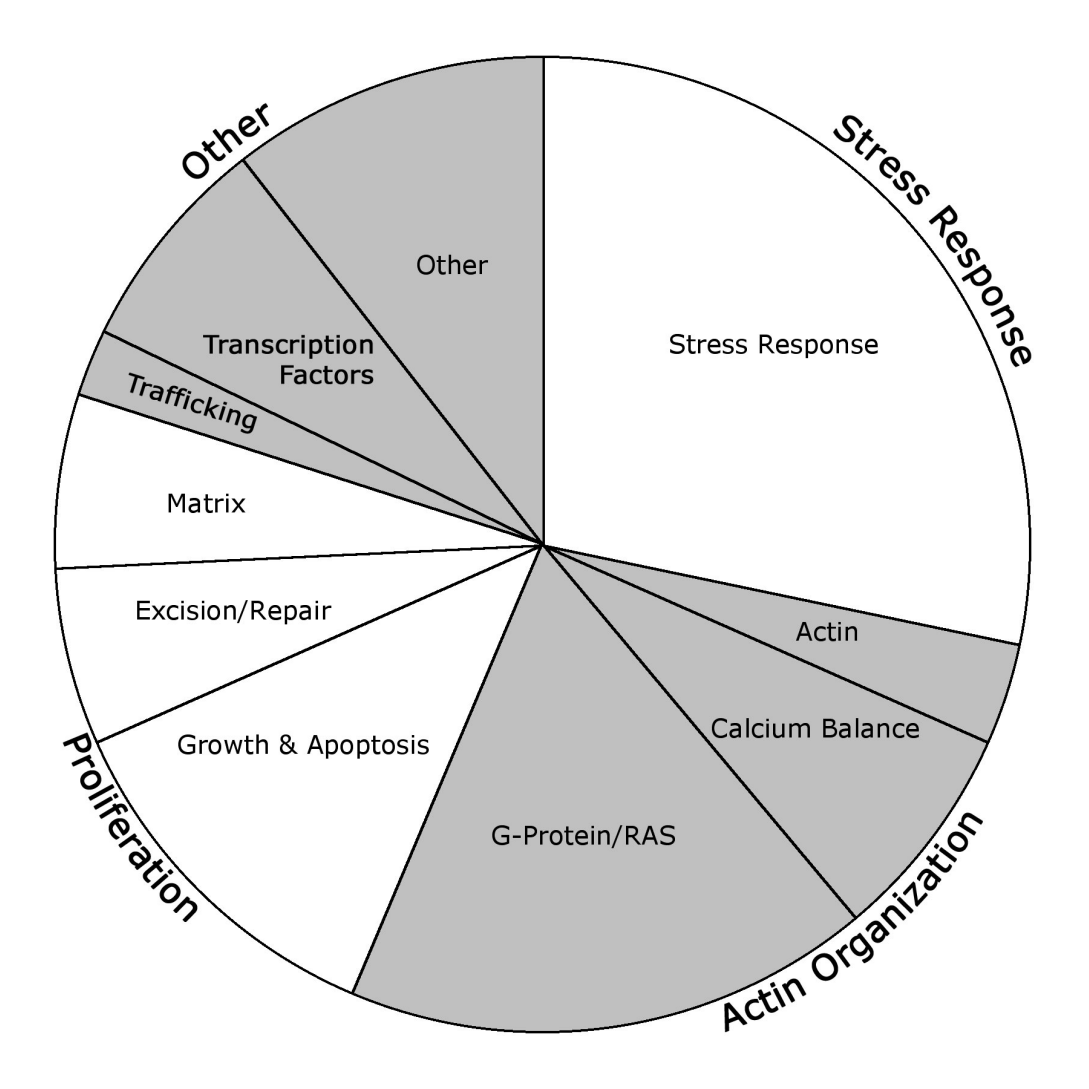
**Ontology grouping of genes differentially regulated in BMPR2 mutation carriers with pulmonary hypertension compared to BMPR2 mutation carriers without disease.** Study design aimed to avoid disease and drug effects, meaning that these should represent predisposition to disease rather than disease effect.

A comparison of unaffected to affected mutation carriers using moderately strict criteria (false discovery rate of 17%) resulted in a list of 80 genes consistently differentially regulated between groups[18]. These fell into several broad groups with known relevance to PAH, including stress response, actin organization, and proliferation (Figure 1, Additional File 1 – altered genes tabulated by group).

### Quantitative RT-PCR Confirms Gene Expression Data

**Figure 2:**
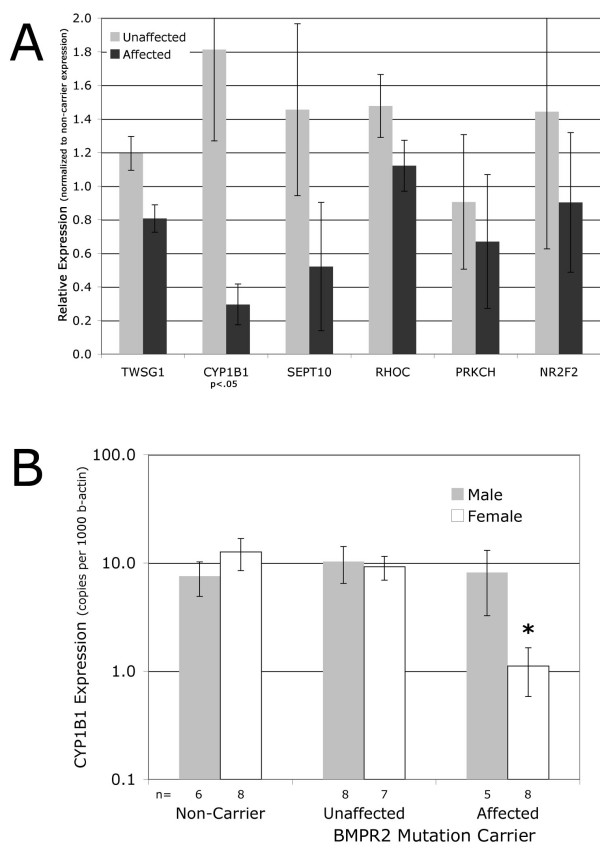
(**A) Expression of selected genes in a larger sample set trends in the same direction as in arrays by quantitative RT-PCR, but both fold-changes and significance are reduced compared to the more limited sample set.** The exception is CYP1B1, in which both fold change and significance are maintained. Expression is normalized to that of beta-actin and then to expression level in non-carriers; p-values are by Kruskal-Wallis. (B) Expression of CYP1B1 is lower in female, but not male, mutation carriers with PAH compared to unaffected carriers and non-carriers. n = number of individuals in category; * = p < .01 for sex-specific effect in disease by two-way ANOVA on log-transformed values.

To confirm relevance of selected specific genes in a larger number of patients, we performed quantitative RT-PCR using freshly derived RNA from 20 lymphoblastoid lines derived from non-carriers, unaffected carriers, and PAH affected carriers from within the same family (using every patient in Table 1). We found that for most genes tested, the direction of change found by array was confirmed in the larger patient sample (Figure 2A). However, the absolute change was somewhat reduced, and for most genes tested the changes were at best trending towards significance. The exception to this was cytochrome P450 1B1 (CYP1B1), which maintained on average greater than a sixfold difference between unaffected BMPR2 mutation carriers and carriers with PAH.

To determine whether decreased CYP1B1 expression was associated with disease status in other FPAH, lymphoblastoid cells were derived from 24 individuals from three distinct patient families, each with too few patients for individual analysis. We found that log-transformed CYP1B1 expression values were significantly decreased (p < .05 by Kruskal-Wallis) in disease-affected mutation carriers compared with unaffected carriers or non-carriers, with a trend towards a sex-specific effect (not shown). When combined with the data from our original family, numbers became sufficient to show a clear sex-specific effect (p < .01 by two-way ANOVA), with CYP1B1 expression levels almost tenfold decreased in female, but not male, affected mutation carriers compared with unaffected carriers or non carriers (Figure 2B).

### Analysis of Overrepresented Gene Ontology Groups Suggests Pathways, not Genes, as Significant Modifiers

There are two explanations for decreasing statistical significance of specific genes with increased samples; the first is that the results are spurious, the second, our hypothesis, was that they are representative of a group, rather than important in themselves.

To test this hypothesis, we focused on the group of GTPases and GTP-binding genes, as defined by the Gene Ontology consortium[20]. These comprise 2179 of the 54613 probes on the U133 Plus 2.0 microarray; when only those genes with sufficient expression to be statistically analyzed are included, they comprise 675 of 12661 probes, or 4.9%. Each of the affected mutation carriers for which we had arrays was compared individually to the arrays from unaffected family member carriers, using the same criteria as before. As expected, given the lower number of comparisons, the number of genes changed in each sample was higher in each sample individually than when they were considered as a group (Table 3, top line), and the false discovery rate increased to a median and average of 38% (determined by mixing group identifiers). This false discovery rate is high; this is why we neither normally attempt statistics on one patient sample nor do we list specific genes found by this method. However, it does not impact subsequent analyses, and keeping the criteria the same for this analysis simplifies comparisons to earlier results.

**Table 3:** GTP-related genes are overrepresented in genes changed in affected BMPR2 Mutation Carriers

	# 723	# 266	# 264	# 186
Genes Changed:	757	386	569	628
Expected GTP-related:	37	19	28	31
Actual GTP-related:	88	54	67	71
z-score	5.4	4.8	4.8	4.7

We found that the number of genes that fell into the GTP-related ontology group was highly significantly overrepresented in each case. If the genes were randomly distributed across ontology groups, we would expect 4.9% of those with altered expression to fall into the GTP-related group (Table 3, second line). Instead, in each case, more than twice as many fell into this group (Table 3, third line), which was highly significant by one-sample z-statistics, with a p < .001 in each case[23].

Next, we considered the significance of the overlap between specific GTP-related genes differentially expressed in each sample. With 625 probe sets changed above the noise and 88 GTP-related genes changed in patient 723, one would expect that 14%(88/625) of the genes changed in each of the other samples would overlap those changed in patient 723. Instead, overlap ranges from 28% to 46% (Table 4 shows numbers of overlapping GTP-related genes between samples), for highly significant overrepresentation of overlap. This shows that it is not all GTP-related genes that are altered in affected BMPR2 mutation carriers, but a specific subset (Additional File 2 lists all GTP-related genes altered in at least two patients).

**Table 4:** Overlap between GTP-related genes changed in different patients

	# 723	# 266	# 264	# 186
# 723	88			
# 266	25	54		
# 264	19	29	67	
# 186	28	28	22	71

p < .001 for overrepresentation for all overlap except #723 vs #264 p = .02

**Figure 3:**
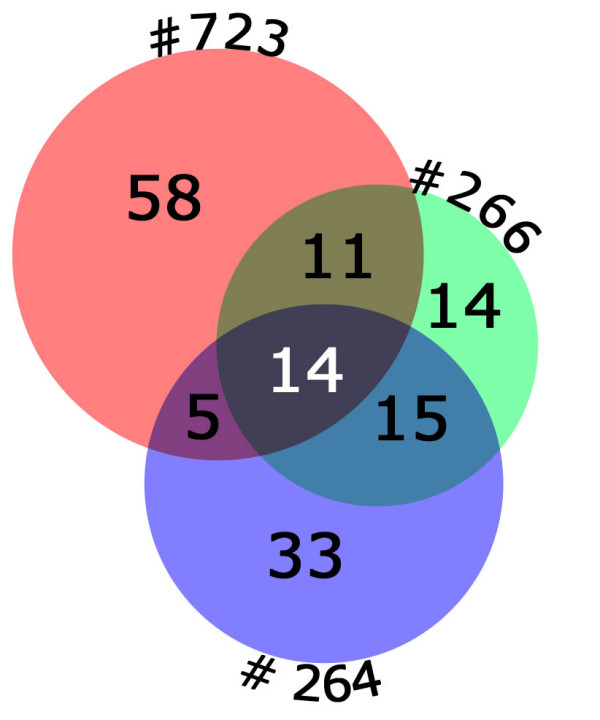
**GTP-related genes have large numbers of differentially regulated genes in all patient samples (registry # is outside of each circle).** Increasing numbers of patients increases the significance of the ontology group, but produces decreasing numbers of genes changed in all patients. For instance, patient #723 and #266 have 25 GTP-related genes in common; this drops to 14 when overlap with patient #264 is added.

Every patient tested has altered regulation of a large number of GTP-related genes, but the number of genes altered in every patient decreases with the number of patients (Figure 3; only three patients are shown for clarity). This analysis suggests that while the significance of specific genes decreases with increased samples, the significance of the group itself increases with increased samples.

## Discussion

The purpose of this study was to identify genes which might predispose BMPR2 mutation carriers to FPAH by comparing carriers unaffected by disease to carriers who developed FPAH. We used cultured B-cell lines in order to remove both disease and drug effects, so that we could examine underlying differences in gene expression.

The strongest individual gene identified by our methodology was Cytochrome P450 1B1 (CYP1B1), in which low expression was associated with disease, but only in women. This gene qualifies as a plausible modifier gene for several reasons. Although we assayed B-cells, CYP1B1 is highly expressed in lung, likely in endothelial cells, and is recognized as a modifier gene for cancers [24-27]. CYP1B1 metabolizes environmental toxins, so it would suggest a plausible mechanism of a gene-environment interaction as a modifier[27]. CYP1B1 also breaks down estrogen, reducing local concentration (systemic estrogen is regulated by other systems) and environmental estrogens are present in modern diets and medications[28]. Since women have a 3:1 overrepresentation in PAH patients, starting at puberty, estrogen is the strongest modifier known for penetrance[29]. Thus, lowered levels of CYP1B1 might result in increased local concentrations of estrogen, further increasing the risk of PAH; that this appears to be a sex-specific modifier gene supports this hypothesis. Follow-up to demonstrate correlation with genetic polymorphisms, thus confirming that this is cause not effect, and a finding of functional consequences in patients are necessary to fully interpret the meaning of this finding.

Our broader finding was that there were consistent differences in regulation of PAH-related pathways between BMPR2 mutation carriers affected and unaffected by disease. These changes are likely related to genetically determined differences in expression, rather than adaptive to disease state or drug effect, because the cells have been cultured to remove them from the immediate environment. Further, B-cells are not believed to unalterably change differentiation state in response to environmental effects. A previous study which examined B-cells freshly derived from patient blood in controls vs IPAH had substantially different findings consisting purely of upregulation of stress-response, suggesting that they were looking at disease and drug effects, and bolstering our belief that disease effects are not a feature of the current study[30].

Pathways changed included stress-response, actin organization, ras-related, g-protein, calcium balance, and proliferation-related pathways (Figure 2). All of these pathways have been previously seen as altered in PAH, but the current study is one of the first to present data suggesting that they are cause rather than effect in human patients[9,31,32]. A detailed analysis of the GTP-related gene ontology group suggested that the important change was in the pathway itself, which could be mediated through different subsets of the genes in the pathway (Figure 3, Tables 3, 4, Additional File 2). This implies that the search for modifier genes may involve either activities or pathways, rather than specific genes. We have seen this effect before; different strains of mice subjected to chronic hypoxia use different genes in the same pathway to achieve similar phenotypic ends[33].

These data are in agreement with our earlier examination of lymphoblastoid cells, in which our strongest candidate was the Ras pathway gene GRB2[34]. Some of the other alterations found in our earlier examination of protein changes in lymphoblastoid cells are also present in this study, but were below the cutoff threshold we selected. For instance, PCMT1, which was increased 1.2× by protein array, was also increased 1.2× by Affymetrix array. Other genes did not have matching changes, likely representing issues of sensitivity and post-transcriptional regulation.

We found more than 80 genes consistently altered between affected and unaffected BMPR2 mutation carriers. It is not probable that there are this many modifier genes; rather, this implies that these genes are downstream of a much smaller number of modifier genes that are having consistent downstream effects. We have already published serotonin transporter(5HTT) and TGF-β promoter polymorphisms as modifier genes contributing to clinical expression in familial PAH, and the types of changes seen are plausible effects for either of these[35,36]. However, we determined that these could not be upstream of the changes seen here, as two of the affected patients had the LL 5HTT promoter and two had the SL promoter. Correlation in gene expression even among altered genes between the two with the LL allele was no stronger than between the LL allele and SL allele (0.82 vs 0.88; correlation between these and unaffecteds averages 0.65 for comparison). Further, these cells do not appear to have a functional TGF-β pathway. Levels of both type 1 and type 2 receptors were below the threshold of detection on the arrays, and both canonical TGF-β target Pai1[37] expression levels and TGF-β luciferase-reporter response were very low (not shown). These data imply that there is a modifier gene upstream of the pathway changes seen, but that it is not a gene currently under consideration.

Our study design carried several inherent limitations. In order to obtain the strongest possible level of discrimination for modifier genes, we compared mutation carriers who developed disease early to mutation carriers still unaffected in old age (Table 1). This necessarily results in a mismatch of age between groups as part of the study design. However, we have previously shown that difference in age does not appear to create a difference in B-cell protein expression[34]. Further, there was a difference in the degree of relation between unaffected and affected carriers; this could result in an underestimate of the false discovery rate. Another limitation lies in differences between B-cells and disease effector cells, including pulmonary smooth muscle and endothelium. For instance, in B-cells, excision/repair (Additional File 1, Figure 1) is tightly linked to proliferation, and so alterations in these genes are probably not meaningful to other cells[15]. Conversely, B-cells do not express some genes likely of interest in disease, and so these cannot be studied in this system. For instance, the TGF-beta system does not appear to be functional in B-cells. However, the ideal tissue comparison, between lung tissue from BMPR2 mutation carriers that will never develop disease and those that will develop disease but have not yet, is inherently impossible; neither the information nor the tissue to conduct this study could be obtained. Finally, the act of EBV-transforming these cells is likely to have produced some changes. However, these changes should be consistent across samples; at worst, they could mask some effects. Concern about this issue explains our focus on GTP-related genes, rather than either stress-response or proliferation, although there is no particular reason to believe that these changes are not also valid.

## Conclusion

The work presented here provides evidence that predisposition to disease in BMPR2 mutation carriers lies in alterations of pathways related to actin organization, stress response, and proliferation, likely achievable through alterations in any of several upstream modifiers, rather than primarily to specific individual genes within those pathways. The strongest single candidate modifier gene identified was CYP1B1, with tenfold lower expression on average in female BMPR2 mutation carriers than in unaffected carriers or non-carriers.

## Competing interests

The authors declare that they have no competing interests.

## Authors' contributions

JW carried out the array and statistical analyses and drafted the manuscript. JC created and maintained the lymphoblastoid lines. MG conducted the array experiments. LR carried out the quantitative RT-PCR experiments. JN participated in the design of the study and collected patient samples. JAP participated in the design and coordination of the study and collected patient samples. KL participated in the design of the study and in data analysis. BM participated in the design of the study and helped to draft the manuscript. JL conceived of the study, and participated in its design and coordination and helped to draft the manuscript.

## Pre-publication history

The pre-publication history for this paper can be accessed here:



## Supplementary Material

Additional file 1 **Genes Differentially Regulated in Affected vs Unaffected BMPR2 Mutation Carriers.** Table, sorted by gene ontology group, of genes differentially regulated between affected and unaffected BMPR2 mutation carriers, including fold changes, absolute values, and associated GO consortium terms. Click here for file

Additional file 2 **GTP-related probes differentially regulated in at least two patients compared to controls. **Table, sorted by gene symbol, of Affymetrix probes differentially regulated in at least two affected BMPR2 mutation carriers compared to unaffected. Values that are differentially expressed at least 1.4× with a minimum 150 absolute change are highlighted in yellow; between 2 and 4 of the affected values for each probe will thus be altered. Click here for file
